# LsrR Quorum Sensing “Switch” Is Revealed by a Bottom-Up Approach

**DOI:** 10.1371/journal.pcbi.1002172

**Published:** 2011-09-29

**Authors:** Sara Hooshangi, William E. Bentley

**Affiliations:** 1 College of Professional Studies, The George Washington University, Washington, DC, United States of America; 2 Fischell Department of Bioengineering, University of Maryland College Park, College Park, Maryland, United States of America; 3 Institute of Bioscience and Biotechnology Research, University of Maryland, College Park, Maryland, United States of America; Duke University, United States of America

## Abstract

Quorum sensing (QS) enables bacterial multicellularity and selective advantage for communicating populations. While genetic “switching” phenomena are a common feature, their mechanistic underpinnings have remained elusive. The interplay between circuit components and their regulation are intertwined and embedded. Observable phenotypes are complex and context dependent. We employed a combination of experimental work and mathematical models to decipher network connectivity and signal transduction in the autoinducer-2 (AI-2) quorum sensing system of *E. coli*. Negative and positive feedback mechanisms were examined by separating the network architecture into sub-networks. A new unreported negative feedback interaction was hypothesized and tested via a simple mathematical model. Also, the importance of the LsrR regulator and its determinant role in the *E. coli* QS “switch”, normally masked by interfering regulatory loops, were revealed. Our simple model allowed mechanistic understanding of the interplay among regulatory sub-structures and their contributions to the overall native functioning network. This “bottom up” approach in understanding gene regulation will serve to unravel complex QS network architectures and lead to the directed coordination of emergent behaviors.

## Author Summary

Quorum sensing is a mechanism by which bacterial cells communicate within a population. One particular form of communication in *E. coli* is through a universal signaling molecule known as autoinducer 2. Although the importance of this form of cell-cell interaction has been recognized in the formation of biofilms and virulent infections, the mechanisms by which this form of communication is regulated is still not well understood. In this paper, we presented a method of unraveling these mechanisms by using a combination of experimental work and mathematical models. We took apart the network architecture and isolated the different components. The examination of these isolated sub-networks provided us with a better understanding of the underlying mechanisms that control and regulate bacterial quorum sensing. We were also able to predict new network interactions with the help of our mathematical models. This bottom up approach, combined with our modeling efforts, proved effective in unraveling the mechanisms of quorum sensing in *E. coli*.

## Introduction

Biological phenomena are frequently controlled by an entangled web of protein and gene networks that constitute regulatory pathways. A large number of such pathways take advantage of environmental cues and signaling molecules to regulate cellular activities. However it is not always clear how biological systems are able to support accurate signal propagation over a sufficiently large dynamic range within the cell. One important factor in determining the fidelity of a signal, in any biological system, is the connectivity of the network, e.g., the interactions among the constituent genes, proteins and metabolites. Recent studies indicate that certain patterns of local connectivity such as negative and positive feedback motifs are more frequently found in natural systems [1], [2].

Despite their individual abilities to influence the flow of information, negative and positive feedback loops are often coupled together in natural systems. One example is the *Xenopus* embryonic cell cycle where a negative feedback loop and a pair of positive feedback loops control the operation of a robust tunable cell cycle oscillator [3]. Several synthetic oscillators have also been built to demonstrate the robust behavior that results from the interaction of various feedback motifs [4]–[6].

In this paper, we examined network connectivity in a natural network which also employs a combination of negative and positive regulation and, at the same time, provides cell-cell communication among bacterial cells. Here, an auto-regulatory network is coupled with a double negative motif to provide a population based response known as bacterial quorum sensing (QS). We examined the network connectivity and followed the propagation of the native signal molecule, autoinducer-2 (AI-2), in this system. Different regulatory motifs of the network were first studied in isolation by constructing mutant strains and were then combined to represent the system as a complete network. Using a combination of experimental work and mathematical modeling, we isolated the mechanisms of AI-2 transport into the cells and investigated how the interplay of the different feedback mechanisms orchestrates the overall cell-cell communication and population based regulation. Our experimental work predicted the existence of a new regulatory element that was not previously suggested. We tested our hypothesis by building a model based on the experimental results that also incorporated the new regulatory element. Our predication was captured well by the model and we were able to simulate both the intact network and all the sub-systems using our new proposed network architecture. Importantly, we revealed the mechanistic basis for the LsrR-mediated genetic “switch” of *E. coli* QS circuitry. In the native system, the switch is buried but still effective. Our modeling results demonstrated the basis for and biological importance of a well-regulated network wherein a balance between the strengths of different feedback motifs is required for the proper functioning of the overall system.

## Results

### The QS mechanism in *E. coli*

**Figure 1:**
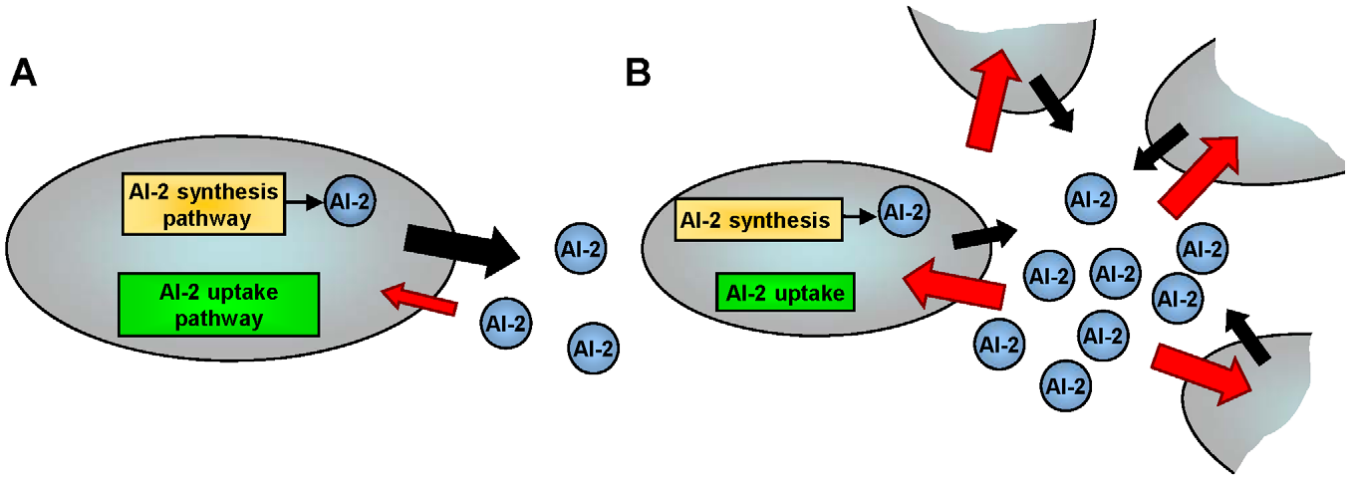
The AI-2 QS mechanism in *E. coli.* During cell growth, AI-2 is synthesized within the cell cytoplasm and transported out of the cell membrane of individual bacterial cells. As the cell density increases, more AI-2 is accumulated within the extra-cellular milieu until the AI-2 concentration reaches a critical “threshold” and at this point it is transported back into the cells.

In general, QS can be described as a density-dependent cell-cell communication process among bacteria that is mediated by the transmission and propagation of chemical signals known as “autoinducers” [7]–[9]. Autoinducers are synthesized within the cell cytoplasm, secreted to the outside and accumulate in the cells’ immediate surroundings [10]. At a point associated with a “quorum” of cells, where the cell density and hence the concentration of the exported autoinducer reaches a threshold, the signaling molecules are transported back into the cells or are bound to cognate cell surface receptors, where they initiate coordinated changes in gene expression [11]. Several classes of signaling molecules and QS mechanisms have been identified [12]. The focus of this work was to investigate the transduction of autoinducer-2 (AI-2) which is the dominate form of cell-cell communication in *E. coli* and *S. Typhimurium*. AI-2 is also suggested to be a “universal” signal molecule due to the presence of its terminal synthase in over 80 genera [8]. Figure 1 summarizes the AI-2 processing mechanism in *E. coli* (for more detailed description, see [13]). During cell growth, AI-2 is synthesized through a multi-step enzymatic pathway and transported out of the cell membrane of individual bacterial cells [14], [15], Figure 1A. The increasing bacterial population results in the accumulation of AI-2 within the extracellular milieu. Once the concentration of AI-2 reaches a critical “threshold” it is transported back into the cell [16], [17], triggering a coordinated genetic response, Figure 1B.

### A “bottom-up” approach reduces the network complexity

Like many biological phenomena, the complexity of the interactions within the QS process, makes the detailed study of this system challenging. We used a “bottom-up” approach to reduce the complexity of the network by separating it into smaller sub-systems (modules) and examining each sub-system in isolation. A more comprehensive picture of the overall network behavior and their interactions could then be elucidated by combining the findings of these modules. We began by separating the AI-2 synthesis/export module from the AI-2 uptake/regulation module shown in Figure 1. Since AI-2 synthesis has been modeled in previous work [13], we focused on understanding the mechanisms of AI-2 transport back into the cell and its transduction/actuation potential. Experimentally, the two modules could be separated by removing one of the AI-2 synthase genes, *luxS*, from the genome and halting the *in vivo* AI-2 synthesis. AI-2 was then synthesized *in vitro* and its concentration estimated by measuring free thiol groups [18]. The synthesized AI-2 was then added to the system at different concentrations and dose response curves were determined. There are two significant advantages to using this *in vitro* approach. First, the separation of these two modules simplifies our analysis by eliminating cross-interaction between *in vivo* synthesized AI-2 and AI-2 that is transported in from the outside. The synthesized AI-2 was treated as an external input added into the system in defined concentrations and hence the exact amount of AI-2 present in the initial system was known. Second, this is a more quantitative approach to study QS mechanisms when compared to studies that rely on measurements of *in vivo* synthesized AI-2. *In vivo* AI-2 studies typically performed using an indirect cell-based assay employing *Vibrio harveyi* bioluminescence [19] which has a high level of day-to-day variability and its irregularities have been noted [20], [21]. As a result, we suggest that our approach is a more quantitative method for the purposes of characterizing network interactions within the QS system.

### Examining the *luxS* knock-out module

**Figure 2:**
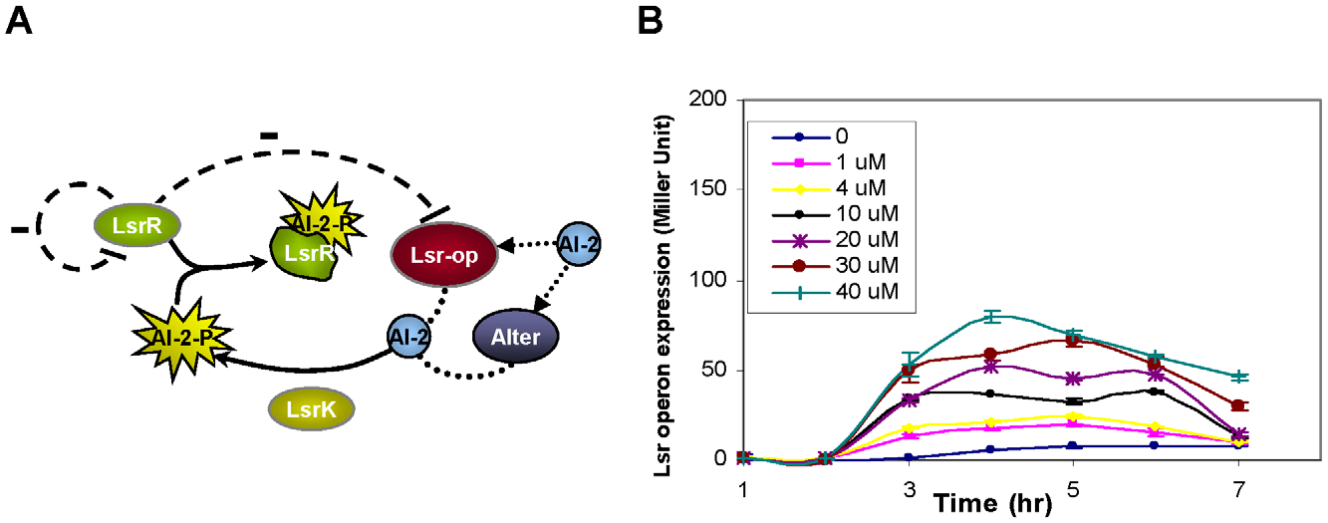
The AI-2 uptake mechanism. A) The previously proposed network architecture of the AI-2 uptake is depicted. AI-2 is transported back into the cell through the transporter Lsr-operon and an unknown mechanism denoted “Alter”. Once AI-2 is inside the cell, it is phosphorylated by LsrK and the phosphorylated AI-2 (AI-2-P) interacts with the LsrR repressor. LsrR is a negative regulator that inhibits both the transcription of the *lsr*-operon and its own. AI-2-P can bind the LsrR protein and prevent the inhibition of the *lsr*-operon promoter. An increase in the levels of the Lsr transporter creates a positive feedback loop where higher concentrations of AI-2 within the cell result in an increase in AI-2 uptake. B) Temporal expression levels of the *lsr*-operon promoter for various concentrations of AI-2 ranging from 0 to 40 µM in *luxS* knock-out strains. Higher AI-2 concentrations result in higher expression rates from the *lsr-*operon. As the cells reach the stationary phase, the expression levels decrease for all doses.

The regulatory elements pertinent to the AI-2 uptake mechanism have been identified in a number of previous studies [15]–[17], [22] and are summarized in Figure 2A. External AI-2 is transported back into the cell through at least one known transporter, Lsr, encoded by the *lsr-*operon. The *lsr*-operon in *E. coli* encodes 6 genes that are responsible for the AI-2 uptake and modification mechanisms. The first four genes (*lsrA*, *lsrC*, *lsrD*, and *lsrB*) encode the import apparatus and the last two genes (*lsF*, *lsrG)* are putative AI-2 processing genes [17], [22] that eliminate the activated form of AI-2 (phospho-AI-2 or AI-2-P) from the cytoplasm in a similar manner to that of *S. Typhimurium* with a highly homologous system [15]. Experimental evidence also indicates that in the absence of the Lsr transporter, AI-2 is can be transported into the cell via an as yet uncharacterized pathway [15]. To account for this unidentified transport mechanism, we included an alternative pathway (denoted “Alter”) in our scheme (Figure 2A). Upon entering the cell, AI-2 is phosphorylated by a cytoplasmic kinase, LsrK, and the phosphorylated AI-2 interacts with a transcriptional regulator, LsrR [23]. LsrR inhibits transcription of the *lsr*-operon by binding to the *lsr*-operon promoter site. LsrR also acts an auto-regulator by binding the *lsrR* promoter site and inhibiting its own transcription [17]. Phospho-AI-2 reportedly binds the LsrR protein and prevents inhibition of the *lsr*-operon, hence alleviating repression and increasing Lsr transporter production. An increase in the levels of the Lsr transporter expedites AI-2 uptake and creates a positive feedback loop where higher concentrations of AI-2 within the cell result in an increase in AI-2 uptake.

Figure 2B depicts the dose response curves for 7 different experiments in which various concentrations of AI-2 were added to growing cultures of *E. coli* cells that lacked the *luxS* gene. Expression levels from *lsr*-operon promoter, which indicate LsrR activity, were measured using a β-galactosidase (β-gal) assay, as described in the Materials and Methods. As expected, higher AI-2 levels resulted in higher expression rates from the operon. As cells reached the stationary phase, the expression levels of all cultures decreased as the *in vitro* AI-2 levels and the cells’ metabolic activity also decreased.

### From single knock-outs to double knock-outs

To better understand the role of each of the regulatory elements presented in Figure 2A and explain the experimental results of Figure 2B, our next step was to systematically remove each of the three key regulatory elements within the AI-2 uptake module and reduced the network into three sub-modules. Each sub-system was constructed by removing either *lsrR*, *lsrK* or *lsr*-operon genes from the genome of strains that already lacked the *luxS* gene. In each of these mutant strains we observed the expression level of the *lsr*-operon promoter as the output. In accordance with previous experimental work [17]) and our experimental observations (data not shown), the deletion of any of these genes has no affect on the cell growth. Once these sub-systems were analyzed in isolation, the individual sub-system responses were compared with the overall network response that was seen in Figure 2B. Our analysis, which is summarized in the following sections, predicted the existence of an additional *lsr*-regulatory mechanism and a new AI-2/protein interaction that had not been hypothesized (or identified) until now.

### Sub-network 1: lsr-operon/luxS knock-out

**Figure 3:**
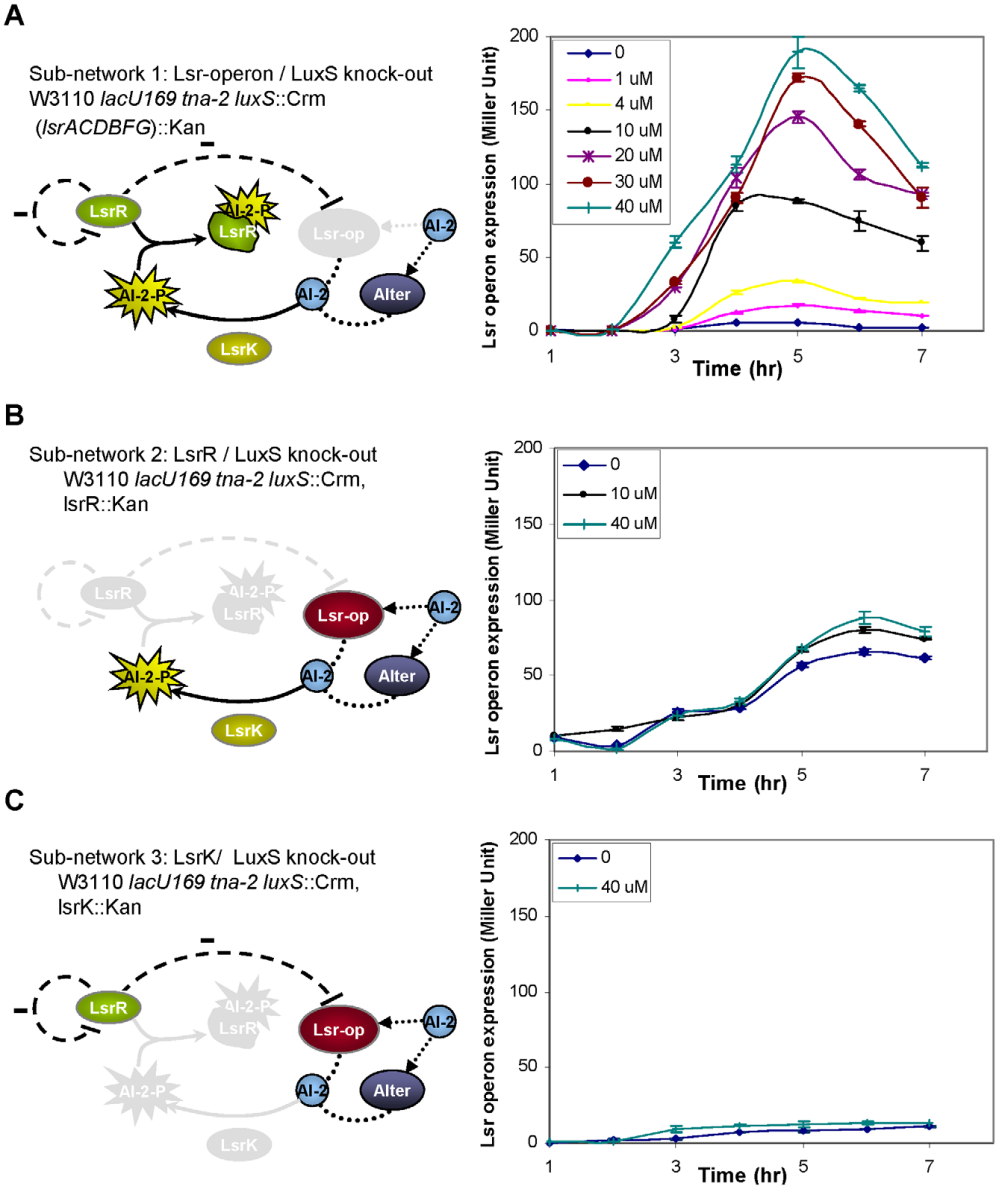
Separating the AI-2 uptake into sub-networks. A) The first sub-network is made by the removal of *lsr*-operon from *luxS* knock-out strains. As the concentration of AI-2 increases, more AI-2-P is available to bind LsrR resulting in higher transcription from the *lsr*-operon promoter. The maximum expression level is reached by hour 5 when most of AI-2 is taken up by the cell. Hereafter, external AI-2 is depleted and the expression levels of all dose responses decline as cells enter the stationary phase. B) The second sub-network is made by the removal of *lsrR* gene from the bacterial genome. The expression of levels of *lsr*-operon is still somewhat dependent on AI-2 despite the fact that there is no LsrR to repress the *lsr*-operon. C) The *lsrK* knock-out represent the third sub-network where in the absence of the LsrK kinase, LsrR completely represses the *lsr*-operon.

The construction of the first sub-system involved the removal of the entire *lsr*-operon from a bacterial strain that already lacked the *luxS* gene, as shown in Figure 3A (experimental details of gene deletion are provided in Materials and Methods). In this sub-network, AI-2 is not transported into the cell via the Lsr-operon transporter but it can still enter the cell using the alternative pathway [19]. The AI-2 which enters the cell via the Alter pathway, is phosphorylated by LsrK (AI-2-P), binds the LsrR protein and prevents the repression of the *lsr*-operon promoter by LsrR. We experimentally monitored the flow of AI-2 by measuring the *lsr*-operon activity over time. Figure 3A shows the temporal responses of *lsr*-operon expression levels from 7 different cultures where various concentrations of *in vitro* AI-2 were added to the growing *E. coli* cultures. As the concentration of AI-2 increases, more AI-2-P is available to bind LsrR resulting in higher transcription from the *lsr*-operon promoter. This accounts for the higher expression levels observed for cultures that are induced with higher AI-2 concentrations. By 5 hr, most of AI-2 is taken up by the cell and the lsr-operon expression reaches its highest level. By 6 hr, external AI-2 is depleted and expression levels decline as cells enter the stationary phase. We observed one surprising result within this sub-system - that the levels of expression were higher without the *lsr*-operon (Figure 3A) compared to what was observed for the intact network (Figure 2B), at the same concentrations of AI-2. The highest level of sub-network 1 was ∼200 Miller units compared to the overall system behavior (∼70 Miller units).

### Sub-network 2: lsrR/luxS knock-out

The second sub-system was built by removing the *lsrR* gene from a bacterial strain that already lacked the *luxS* gene (shown in Figure 3B). Without the presence of the repressor protein, LsrR, the *lsr*-operon is not repressed and AI-2 can enter the cell via both the *lsr*-operon and the alternative pathway. According to this model depiction, the expression level of the *lsr*-operon is independent of the AI-2 levels and we expected to see high levels of expression from the *lsr*-operon irrespective of the concentration of AI-2. Figure 3B represents the time response of three cultures grown with different levels of AI-2. Contrary to our prediction, the expression levels were not as high as, nor higher than, those seen in Figure 3A. Further, the system appeared to have a slight dependency on AI-2 concentration as higher levels of expression were found for higher AI-2 concentrations.

### Sub-network 3: lsrK/luxS knock-out

The last sub-system was built by removing the *lsrK* gene from the isogenic parent strain. Without the LsrK protein and the corresponding AI-2-P in the system, LsrR is free to fully repress the *lsr*-operon. Therefore, we expected that the *lsr-*operon expression levels should be low regardless of input AI-2 concentrations. Figure 3C represents the experimental data for 2 samples at both low and high concentrations of AI-2. In agreement with our expectations, the expression levels were both low.

### A simple mathematical model of the QS network

The result shown in Figure 3A and 3B indicated that the *lsr*-operon mutant (Figure 3A) had higher expression levels than the parent strain (Figure 2B) and that *lsrR* mutant response (Figure 3B) had a slight dependency on AI-2 concentration (Figure 3B). These observations suggested that the perceived network architecture as presented in Figure 2A might not be an accurate depiction of the overall system behavior. Other regulatory elements and possible feedback mechanisms, not identified in previous studies, may be involved in the AI-2 uptake/transduction process.

In order to account for such regulations, we constructed a mathematical model of our network and its sub-systems. We started by modeling the AI-2 uptake by the cell and its downstream regulation that were depicted in Figure 2A. Table 1 and 2 summarize the details of this first model. The synthesis of LsrR and Lsr-operon were each modeled by a single equation (Eq.1 & 2, Table 1) where protein synthesis, decay, cooperative binding and repression were all encompassed in a single equation. AI-2 delivery to the cell was modeled by an active transport of AI-2 by the Lsr-operon and also an alternative pathway that was represented by a simple flux (Eq. 3, & 4, Table 1). Our experiments have indicated that the presence of LsrK is essential to the operation of this network and that in the absence of this kinase the system is shut down. Moreover a kinetic analysis of LsrK activity in a recent paper [24] has shown that the phosphoralytion of AI-2 is rapid and is completed within a few minutes. As a result, to simplify the network description we made the assumption that all the imported AI-2 was phosphorylated (denoted as Ap in the model). AI-2 interaction and binding to LsrR was modeled as a formation of a complex and its eventual decay (Eq. 5, Table 1). Initial concentration of all variables, except AI-2, was set to zero (Table 2). AI-2 was modeled as an input to the system and its concentration varied over a range of 1 to 40 uM to match the *in vitro* AI-2 concentration used in our experiments.

**Table 1:** Ordinary differential equations used in the first model.

Reaction	Differential equation	
Lsr-operon synthesis	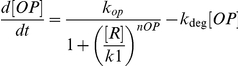	(1)
LsrR synthesis	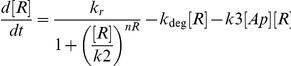	(2)
AI-2 inside the cell	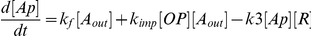	(3)
AI-2 outside the cell	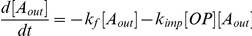	(4)
AI-2/LsrR interaction	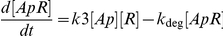	(5)

The system of ordinary differential equations used to model the system is described here. This is based on the network topology depicted in Figure 2A.

**Table 2:** Model species and kinetic parameters used in the first model.

Species	Description	Initial Condition/Range
t	Time	[0, 500] min
OP	Lsr-operon concentration	0 M
R	LsrR concentration	0 M
Ap	Phospholyrated AI-2 within cell	0 M
Aout	AI-2 concentration outside the cell	[1], [40] uM

Parameters	Description	Best fit value (first model)
k_op_	Lsr-operon synthesis rate	2.3 uMol^−1^ min^−1^
k_r_:	LsrR synthesis rate	2 min^−1^
k1:	Repression coefficient (Lsr-operon)	0.2 uMol
k2:	Repression coefficient (LsrR)	0.1 uMol
k3:	AI-2/LsrR binding rate	0.05 uMol^−1^ min^−1^
k_f_	AI-2 flux for the alternative pathway	0.0001 uMol^−1^ min^−1^
k_imp_:	AI-2 import rate by Lsr-operon	0.0005 uMol^−1^ min^−1^
nOP:	Cooperativity coefficient (Lsr-operon)	4
nR	Cooperativity coefficient (LsrR)	4
k_deg_	Protein decay	0.02 min^−1^

Summary of the initial concentration of the species in the first model and the complete list of kinetic rates that provided the best fit to the experimental data shown in Figure 2B.

A deterministic solution to the system of ordinary differential equations presented in Table 1 was evaluated using the freely available software COPASI [25]. In the deterministic framework, COPASI calculates time course by using a LSODA integrator [26] that will numerically evaluate a solution to the system. Since few empirical data on the kinetic parameters of this system was available, a parameter estimation routine was used to fit and match the model to the experimental data shown in Figure 2B. A global parameter estimation routine based on a least-squares method and a direct search algorithm [25], [27] was used to minimize the distance between experimental data and this first model. The parameterization routine (the range of parameters are listed in Table S3) was performed on all the kinetic parameters that are listed in Table 2 with the exception of protein decay which was set to cell division time. This assumption is valid as the proteins in this network are generally stable and have half-lives that are much larger than the cell division time [17]. The cell division time was set to 30 minutes based on previous experiments performed in our laboratory and the understanding that mutations do not affect cell growth [17], [22]. Table 2 lists the kinetic parameters that provided the best correlation to the experimental result of Figure 2B. Figure S1 represents the dynamic response of this corresponding first model for 10 AI-2 concentrations as described in Text S1. As it is evidence from these graphs this model is a good representation of the experimental data.

We then used the same model parameters to simulate the response of the sub-systems that are presented in Figure 3. The result of these simulations (time responses are shown in Figure S2) was not a good representation of the experimental data. In the model, in the absence of Lsr-operon, the AI-2 uptake is significantly impaired and LsrR is able to repress the operon expression level to very low levels. Since our experimental results of Figure 3A were unexpected, it was not a surprise that the model also did not reproduce the experimental data and that we were unable to simultaneously reproduce the experimental results observed in Figure 2B and Figure 3. A global parameter estimation routine revealed that, using the same model description, several parameters such as *k1*, *k2* and *nR* needed to be significantly changed in order to reproduce Figure 3A or 3B alone.

In order to rectify these discrepancies, several different network topologies were considered as possible alternative configurations for this system. For example the Lsr-operon transport and the alternative pathway might work as mutually exclusive switches where in the presence of Lsr-operon the alternative pathway is shut down. There is also the possibility that LsrR has other regulatory roles in the network that are not well understood. One interaction that was hypothesized and showed promise in our preliminary evaluations was the existence of a second regulatory mechanism (in addition to LsrR) that controls the transcription of the *lsr*-operon. In theory, an *lsr*-regulator protein may act as a negative regulatory mechanism to repress the activity of the *lsr*-operon promoter site. The original model (denoted first model) was modified to include this interaction. The modification involved introducing a protein named “REG” as a second repressor in the system and adding a repression term within the Lsr-operon synthesis equation. A new parameter estimation routine was performed to correlate the model with both the experimental result shown in Figure 2B and Figure 3 simultaneously. The new model allowed us to replicate the experimental results in Figure 2B and 3A quite well (time courses shown in Figure S3 and explained in Text S1) but it did not support the weak dependency of *lsr*-operon expression on AI-2 that was demonstrated in Figure 3B.

**Figure 4:**
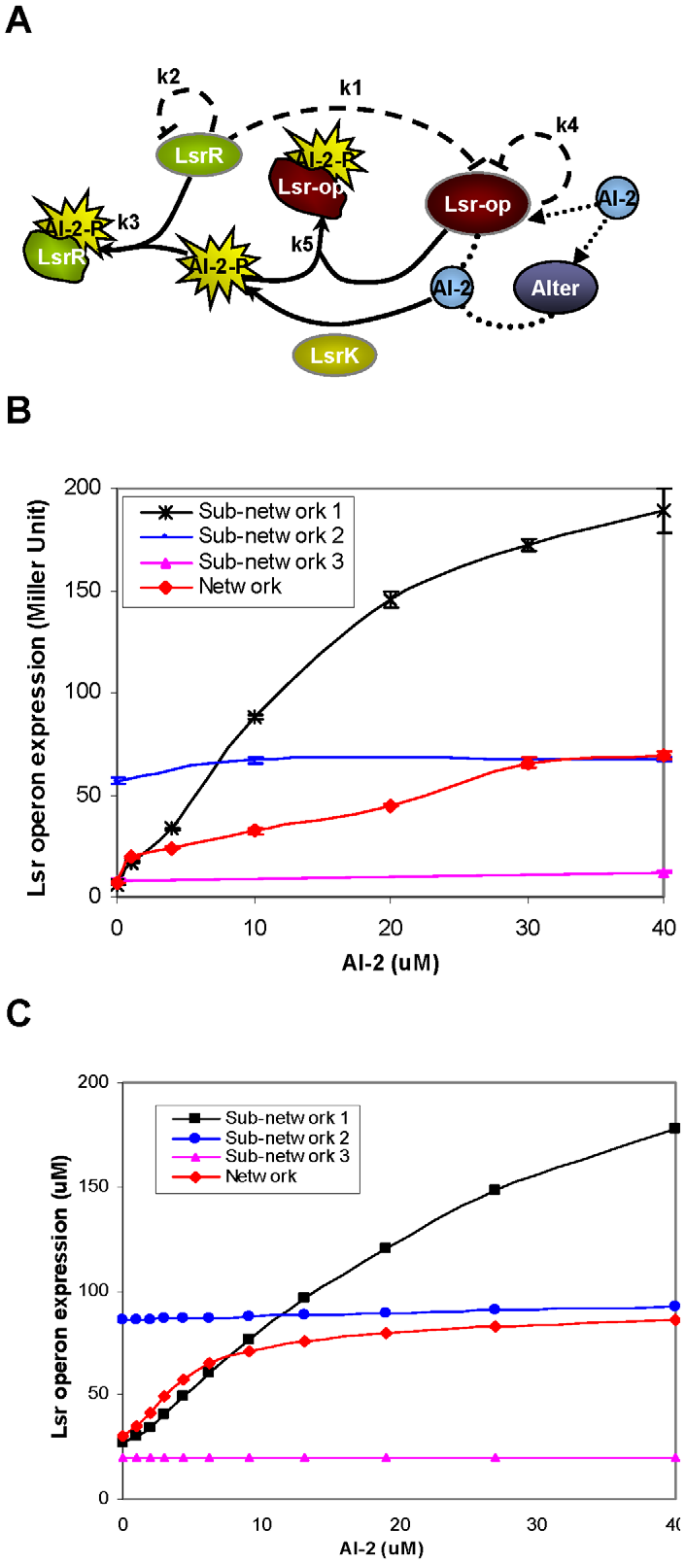
Modifying the network architecture. A) A new model representation of the AI-2 uptake mechanism is proposed. Here REG protein acts a negative regulator to repress the expression of the *lsr*-operon promoter. In the presence of high AI-2 concentrations, REG (like LsrR) binds AI-2-P and the auto-regulation effect of REG decreases. B) The *lsr-*operon promoter activity is plotted as a function of AI-2 concentrations at a single time point (hour 5) for all three sub-networks (*lsr*-operon, *lsrR* and *lsrK* knock-outs) and also for the intact network. C) Simulation results with the appropriate parameter sets show close correlation of the proposed model to the experimental work.

We looked for other possible scenarios that could account for this apparent weak dependency on AI-2 in the absence of LsrR. One possible explanation, as shown in Figure 4A, is that the *lsr*-regulator [28], similar to LsrR, could bind AI-2-P and reduce the repression of the *lsr*-operon in the presence of high concentrations of AI-2. This could result in higher expression levels from the promoter in the absence of *lsr*-operon (seen in Figure 3A) and an increase in expression levels with the addition of AI-2 (seen in Figure 3B). This is conceptually feasible, as described later, as REG protein processing can both eliminate AI-2-P as a positive regulator over long periods of time and it can also sequester AI-2-P while it is bound.

Using the described network architecture (Figure 4A) we were able to produce simulation results that matched all our experimental results closely (see Figure S4 for time response simulations). Table 3 describes the new set of equations in this modified model and Table 4 lists all the kinetic parameters that were used.

**Table 3:** Modified model description.

Reaction	Differential equation	
Lsr-operon synthesis	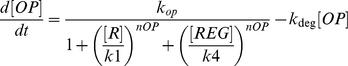	(1)
Lsr-regulator synthesis	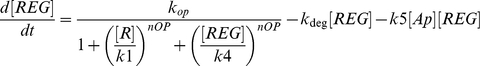	(2)
LsrR synthesis	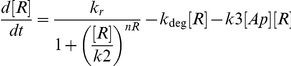	(3)
AI-2 inside the cell	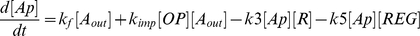	(4)
AI-2 outside the cell	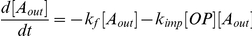	(5)
AI-2/REG interaction	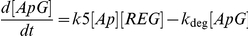	(6)
AI-2/LsrR interaction	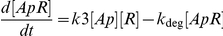	(7)

Modified model that includes both protein REG regulation and also REG/AI2-P interaction.

**Table 4:** Kinetic rates used in the modified model.

Parameters	Description	Best fit value (modified model)
k_op_	Lsr-operon/Lsr-regulator synthesis rate	7 uMol^−1^ min^−1^
k_r_:	LsrR synthesis rate	2 min^−1^
k1:	Repression coefficient (Lsr-operon)	0.2 uMol
k2:	Repression coefficient (LsrR)	0.1 uMol
k3:	AI-2/LsrR binding rate	0.05 uMol^−1^ min^−1^
k4	Repression coefficient (Lsr-regulator)	65 uMol
k5	REG/AI2 interaction	0.0001 uMol^−1^ min^−1^
k_f_	AI-2 flux for the alternative pathway	0.01 uMol^−1^ min^−1^
k_imp_:	AI-2 import rate by Lsr-operon	0.01 uMol^−1^ min^−1^
nOP:	Cooperativity coefficient (operon)	4
nR	Cooperativity coefficient (LsrR)	4
k_deg_	Protein decay	0.02 min^−1^

The list of kinetic rates used in the modified model. Two new parameters (k4 and k5) are introduced in the modified model.

In order to show the comparison between the model and experiments more clearly we plotted the promoter activity as a function of the AI-2 concentrations at a single time point (time of maxima in simulations shown in Figure S4 and experiments shown in Figure 2 and 3) as shown in Figure 4B and 4C respectively. This representation captures the effect of AI-2 on individual sub-networks and at the same time allows us to compare the different sub-networks within the network. Our simulation results matched our experiments closely and indicated the possible existence of the interaction between the *lsr*-operon, its own promoter and the AI-2-P.

### *In vitro* vs *in vivo* AI-2

**Figure 5:**
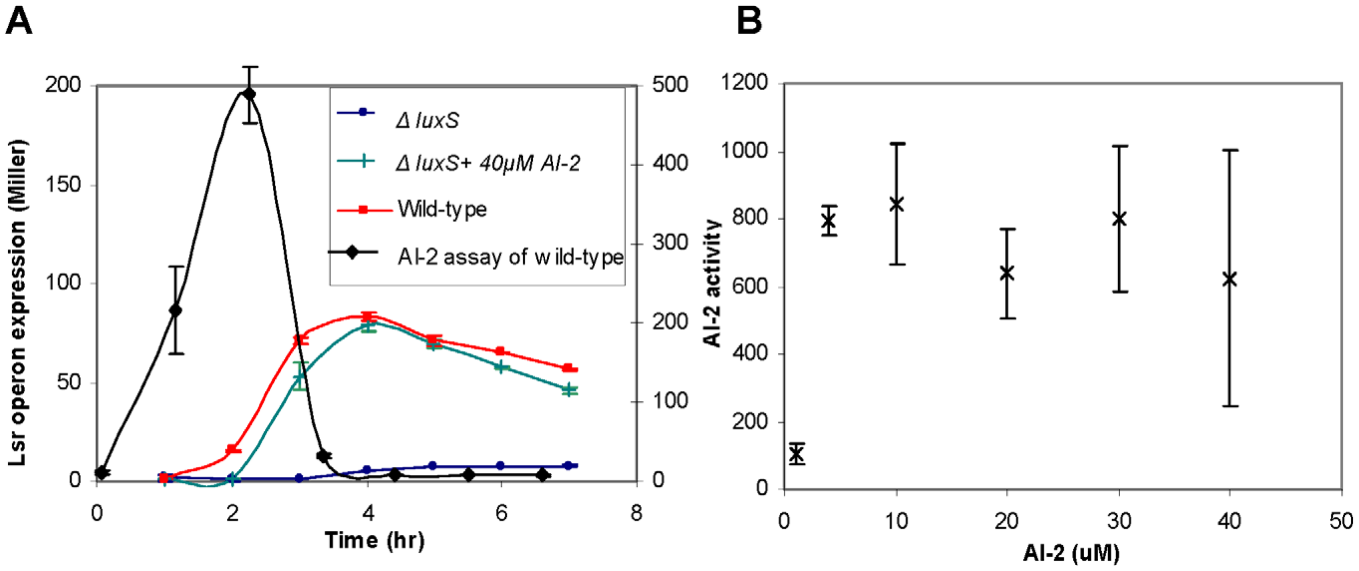
The effects of *in vitro* vs *in vivo* AI-2. A) A comparison between the *lsr*-operon expression level of *luxS* mutant cells (−/+ AI-2) with the wild-type expression levels. Similar expression levels were observed between high AI-2 (green line) and the wild-type (red line). Black line (measured on the second y-axis on the right) represents the AI-2 import profile for the wild-type strain. B) *In vitro* AI-2 activity was measured using the *Vibrio harveyi* assay to find the corresponding AI-2 concentration that has similar effect as the wild-type. AI-2 level of around 4 µM give similar activity level as the wild-type response.

After a close examination and deciphering of the AI-2 uptake mechanism it was interesting to go back and compare our results with the responses of wild-type *E. coli*. We compared the *lsr*-operon expression level of *luxS* knock-out cells (−/+ *in vitro* AI-2) with the wild-type expression levels. Expression levels for the wild-type and the high *in vitro* AI-2 supplemented cases were similar (Figure 5A, left y-axis). For reference, we depicted the measured AI-2 activity of *V. harveyi* within the extracellular milieu of the cultivated wild-type cells on the same graph (Figure 5A, right y-axis). As expected, AI-2 is accumulated and upon reaching a threshold, it is transported back into the cell. The highest level of AI-2 activity for the wild-type cells, measured through the AI-2 assay (see Materials and Methods), was around 200 units of luminescence. We then measured the activity levels of different *in vitro* AI-2 concentrations to find the level of *in vitro* AI-2 that yielded expression levels as that in the *in vivo* synthesized AI-2 case (Figure 5B). To our surprise, we noticed that an *in vitro* concentration of only 4 µM gave similar AI-2 activity as the wild-type. For the higher *in vitro* AI-2 concentrations we observed greater luminescence (around 1,000 units of luminescence). According to our results in Figure 2B, 4 µM of *in vitro* AI-2 can only marginally de-repress the *lsr-*operon and the expression levels of the promoter in this case are much lower than what is seen in the wild-type strain. This is a very important observation as it points out the possibility that AI-2 might not be completely exported out of the cell in the wild-type cells and that the internally made AI-2 might also contribute to the regulatory mechanisms of AI-2 uptake [17], [29]. Furthermore, in addition to being a signaling molecule, AI-2 might have other regulatory and metabolic roles within the cell. Further investigation of the internal AI-2 is required to shed light on some of these observations.

## Discussion

The AI-2 uptake mechanism, which is one of the two main modules in the AI-2-mediated QS system in *E. coli*, was taken apart in this work and the sub-systems were analyzed in isolation. We separated our original network architecture into simpler modules, examined each module separately and this led to the speculation of new regulatory interactions within the network. Several different interactions including the existence of other active transports, a mutually exclusive switch like behavior of the Lsr-operon transport and the alternative pathway and enzymatic activity of other proteins that might degrade AI-2 were all considered and examined in a preliminary test. One scenario that showed promise was the case in which an *lsr*-regulator protein would interact with the *lsr*-operon promoter site and act as a negative regulator (based on the results obtained in Figure 2B and 3A). In addition the response of the *lsrR* knock-out strains (Figure 3B) suggested a possible interaction of the *lsr*-regulator protein with AI-2-P. These new interactions were incorporated into the construction of a comprehensive system of ordinary differential equations, details of which are summarized in Tables 3–4.

Four types of regulations played an important role in determining the outcome of this model i) the LsrR repression of the *lsr*-operon (represented by *k1*), ii) the auto-regulatory effect of LsrR on its own promoter sites (represented by *k2*), iii) interaction of *lsr*-regulator (REG protein) with the *lsr-*operon promoter site (represented by *k4*), and iv) AI-2-P binding to LsrR and REG (represented by *k3* and *k5*). The regulation of LsrR and REG (negative feedback motifs) were coupled with the AI-2-P binding of the LsrR and REG (where the binding acts as de-repressing mechanism and hence has an overall positive feedback effect) to give this network a combination of both negative and positive feedback regulation. Our modeling results indicated that only with in a specific range of kinetic rates we were able to produce the desired response that correlated well with all the experimental results. The best correlation between the model and the experiments occurred when the relative affinity of the LsrR for the *lsrR* promoter was of the same order as the LsrR affinity for the *lsr*-operon promoter. This indicates that LsrR has similar binding affinity for its own promoter and the *lsr*-operon promoter site and that a competitive dynamics exists within this network architecture to bind LsrR repressor protein. The degree of regulation of the *lsr*-operon by REG protein was less than the LsrR auto-regulation strength and the binding affinity of AI-2 for the *lsr-*regulator was also lower than its affinity for the LsrR protein. This indicates that LsrR plays a more significant role in regulating the dynamics of the QS network suggesting that this protein might be involved in other metabolic pathways within the cell as speculated by others [29]. Our further analysis of the system revealed that the *in vitro* AI-2 levels required to produce similar effects on the system are very high compare to what is expected based on the secretion of *in vivo* AI-2. This observation suggests that AI-2 might have other regulatory roles within the cells that are still unknown.

The two most important revelations in this work are: the apparent negative feedback regulation on the *lsr*-regulator that is as strong as or stronger than that of the LsrR protein, and the uncovering of the strength of the Lsr “switch”. Negative regulation is a recurring motif in biological networks and previous works have shown that this motif is able to reduce transcriptional noise in single genes and cascades [30]–[32] and increase the fidelity of signal transmission in biological networks [33], [34]. In our case, the mechanistic basis for the *lsr*-regulator’s effect on repression is only partially explained. The regulator’s processing of AI-2-P would understandably sequester and reduce AI-2-P, enabling higher transcription of the *lsr*-operon. However, our conjecture that an *lsr*-regulator is repressing the promoter site via direct binding or other mechanism is supported by several currently disjoint observations. First, we found LsrR binding to both the *lsrR* and *lsr*-operon promoters were predicted to be similar in strength. This suggests coordination between LsrR proteins, which would be feasible if LsrR operates as a dimer [35] and these sites are brought into proximity. Additionally, since the data from the *lsr*-operon mutant are significantly higher than the *lsrR* mutant, the net effect should be the recruitment of other factors, not currently considered. We hypothesize two such scenarios: first, we have shown here the effects of *in vivo* AI-2 are significantly different than imported and phosphorylated AI-2. We have also demonstrated that unphosphorylated AI-2 plays a role in modulating LsrR regulated gene expression [29]. In the *lsr*-operon mutant, AI-2 is transported into the cell via alternative pathway, presumably phosphorylated by LsrK, but not degraded by LsrFG. This will alter the relative ratio of AI-2 and AI-2-P which might influence *lsr* expression in an as yet undetermined manner. Second, in ours [17] and Bassler’s previous report [16] there is significant interplay between CRP and *lsr* expression, and binding sites in the intergenic region were revealed. Perhaps the *lsr*-operon and the AI-2-P state alter the effects of CRP in this regulatory switch. We note that the presence of glucose completely shuts off *lsr* expression and swamps the QS regulation mediated by LsrR [17]. The second most important revelation was that the LsrR “switch”, in fact, is significantly stronger when turned on in sub-networks than in the wild-type cells. In effect, we have found the levels of AI-2 needed to toggle this switch and hypothesize that it might be of use in guiding phenotype. Switch-like behavior is a common phenotype of network topologies that have some degree of positive regulation or feedback [36], [37] and in our case the binding of the repressor protein to AI-2-P effectively plays this role and is the further evidence of the existence of a switch.

Our work is the first study of QS wherein a combination of double knock-outs and *in vitro* synthesized AI-2 have been used to quantify gene regulation in the *E. coli* AI-2 system. We showed that using a bottom-up approach and isolating the important regulatory elements is an effective way to analyze a natural biological network especially when positive and negative regulations exist within the network architecture. We also showed that the study of such isolated modules allows one to construct a hypothetical model of the system and use simulations to predict the existence of new regulatory mechanisms. Further analysis of this network architecture will shed light on other regulatory pathways within the metabolic networks of bacterial cells and may be used to guide phenotypes in new ways as the quorum sensing switches become incorporated into various biotechnological applications.

## Materials and Methods

### Bacterial strains and growth media

The bacterial strains used in this study are listed in Table S1. Luria-Bertani broth [38] contained 5 g of yeast extract (Sigma) liter^−1^, 10 g of Bacto tryptone (Difco) liter^−1^, and 10 g of NaCl liter^−1^. Media were supplemented with antibiotics at the following concentrations: Ampicillin, 20 µg ml^−1^; Kanamycin, 10 µg ml^−1^ and Chloramphenicol 10 µg ml^−1^.

### Chromosomal deletions of *lsrR*, *lsrK*, and the *lsrACDBFG* operon

The one-step replacement method described by Datsenko and Wanner [39] was used to construct a *luxS* deletion in *E. coli* strains LW8, LW9 and LW11. The phage λRed recombination system was used to replace the *luxS* gene with a luxS::Crm PCR fragment. pKD3 plasmid was used as PCR template with primers luxSHP1 and luxSHP1 (Table S2). The PCR products were then treated with DpnI and introduced by electroporation into *E. coli* LW8, LW9 or LW11 strains containing pKD46 plasmid. The strains were then grown in 37°C for an hour. Recombinants were selected on LB plates supplemented with Kanamycin and Chloramphenicol. The deletion of the genes was verified by PCR tests.

### ß-Galactosidase assays

Cultures of *E. coli* were grown overnight in LB, diluted 100-fold into fresh LB, grown to the OD600 below 0.05. The cultures were incubated at 37°C with shaking at 250 rpm and grown for 1 hour. AI-2 was then added to the system and samples were places back in the incubator. Samples were removed at hour intervals for determination of the OD600 and β-galactosidase activity using the Miller method [40]. The Specific activity of β-galactosidase was expressed in Miller units [40].

### AI-2 activity assay

Cell-free culture fluids were prepared by centrifugation of the *E. coli* samples culture at 10,000 rpm for 5 min in a microcentrifuge. Cleared supernatants were filtered (0.2 µm size HT Tuffryn filters; Pall Corp., Ann Arbor, Mich.) and stored at −20°C. These cell-free culture media were tested for the presence of AI-2 by inducing luminescence in *Vibrio harveyi* reporter strain BB170. The assays were performed as outlined by [41].

## Supporting Information

Figure S1 **Time response of Lsr-operon.** Lsr-operon dynamics for different AI-2 concentrations is simulated and presented.
(TIFF)

Figure S2 **Time response of the subsystems.** The simulation result for subsystems. *lsr*-operon knock-out (left) and *lsrR* knock-out (right) is depicted and compared.
(TIFF)

Figure S3 **Time response of the subsystem for the modified model.** The simulation result for the second model that includes the *lsr*-operon regulator. Intact network (left) and *lsr*-operon knock-out (right).
(TIFF)

Figure S4 **Time response of the final model.** The simulation result for the third model. Intact network (left) and *lsr*-operon knock out (right).
(TIFF)

Table S1 **Plasmids and strains.** Bacterial strains and plasmids used in this study are listed.
(TIFF)

Table S2 **Primers.** Primers used in this study have the following sequences.
(TIFF)

Table S3 **Parameter ranges.** Range of parameters that were explored during the parameter fitting process is listed in this table.
(TIFF)

Text S1 **Model and simulation results description.**
(PDF)
