# Supplementary figures and images for: LsrR Quorum Sensing “Switch” Is Revealed by a Bottom-Up Approach
Source: PLoS Comput Biol. 2011 Sep 29;7(9):e1002172. doi: 10.1371/journal.pcbi.1002172 (PMC3182856; doi:10.1371/journal.pcbi.1002172)

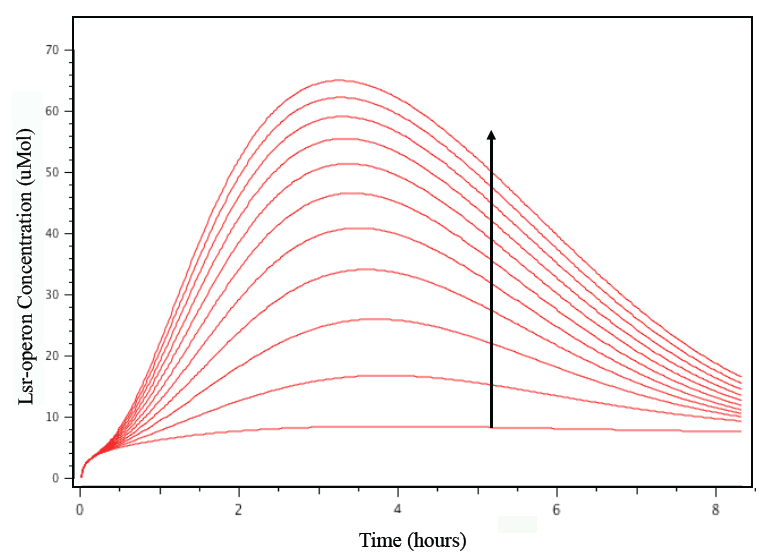

Supplement: Figure S1 — Time response of Lsr-operon. Lsr-operon dynamics for different AI-2 concentrations is simulated and presented. (TIFF) [file pcbi.1002172.s001.tiff]

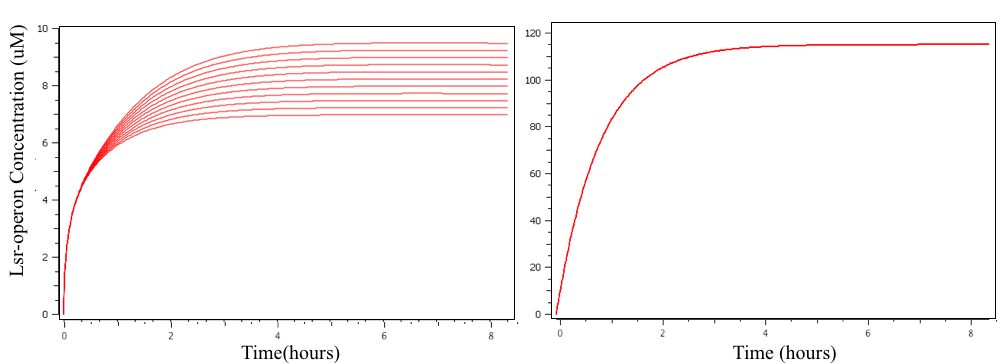

Supplement: Figure S2 — Time response of the subsystems. The simulation result for subsystems. lsr-operon knock-out (left) and lsrR knock-out (right) is depicted and compared. (TIFF) [file pcbi.1002172.s002.tiff]

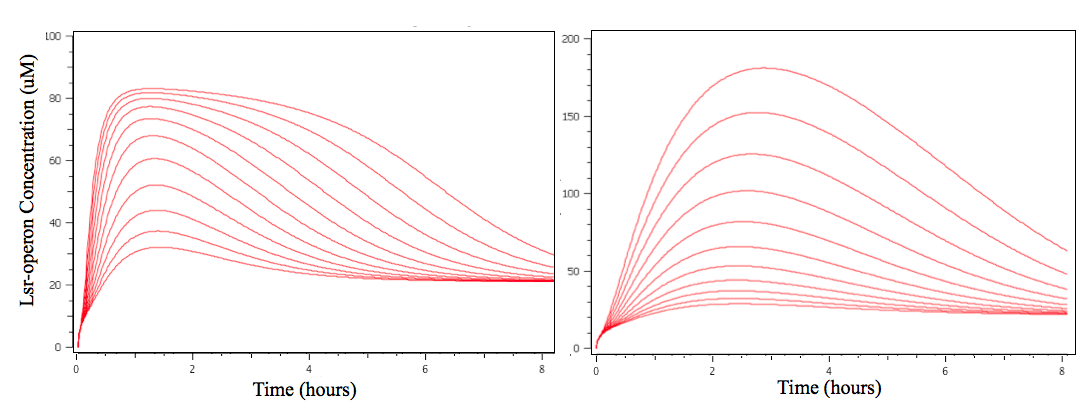

Supplement: Figure S3 — Time response of the subsystem for the modified model. The simulation result for the second model that includes the lsr-operon regulator. Intact network (left) and lsr-operon knock-out (right). (TIFF) [file pcbi.1002172.s003.tiff]

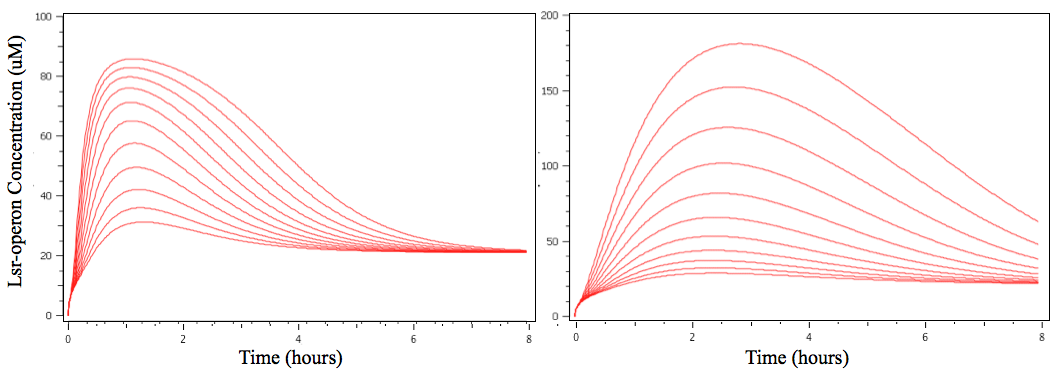

Supplement: Figure S4 — Time response of the final model. The simulation result for the third model. Intact network (left) and lsr-operon knock out (right). (TIFF) [file pcbi.1002172.s004.tiff]

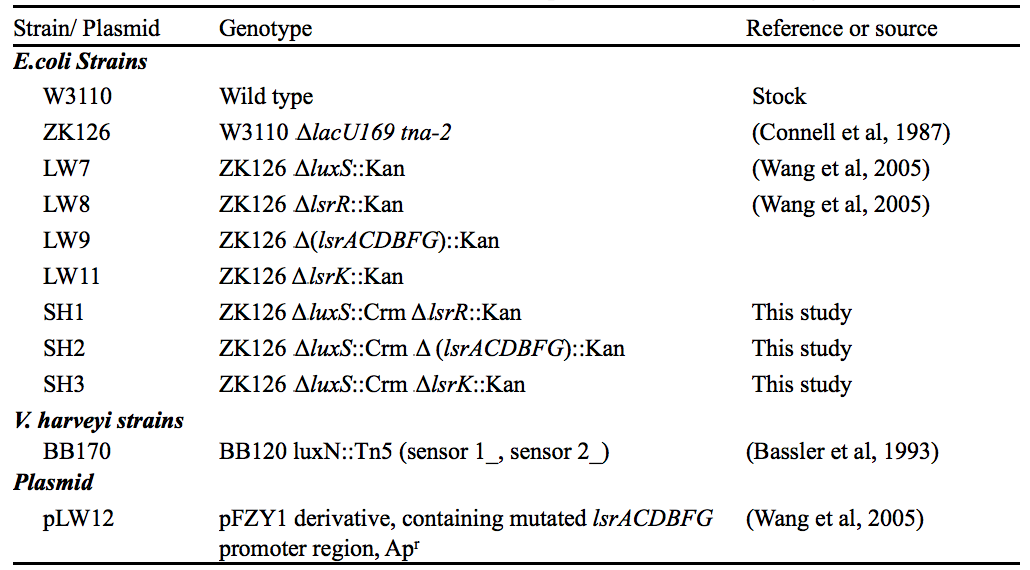

Supplement: Table S1 — Plasmids and strains. Bacterial strains and plasmids used in this study are listed. (TIFF) [file pcbi.1002172.s005.tiff]

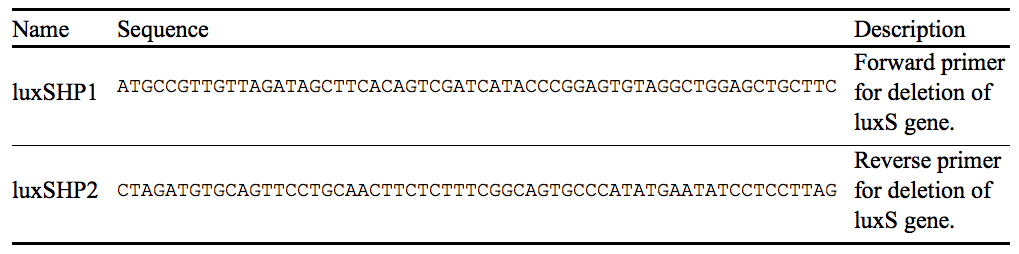

Supplement: Table S2 — Primers. Primers used in this study have the following sequences. (TIFF) [file pcbi.1002172.s006.tiff]

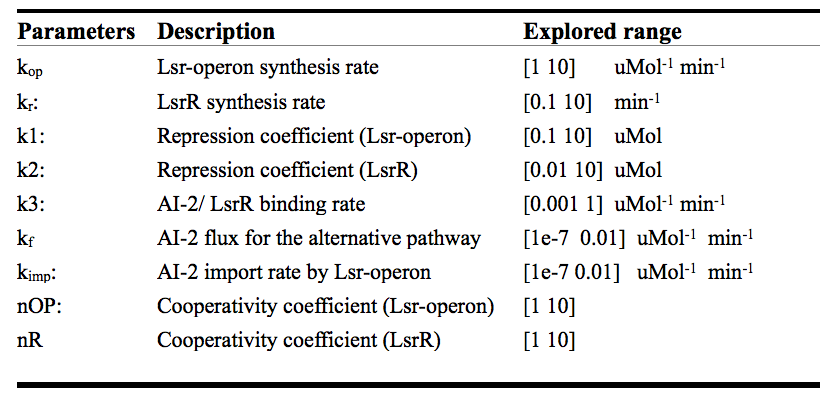

Supplement: Table S3 — Parameter ranges. Range of parameters that were explored during the parameter fitting process is listed in this table. (TIFF) [file pcbi.1002172.s007.tiff]
