# Supplementary material for: LsrR Quorum Sensing “Switch” Is Revealed by a Bottom-Up Approach
Source: PLoS Comput Biol. 2011 Sep 29;7(9):e1002172. doi: 10.1371/journal.pcbi.1002172 (PMC3182856; doi:10.1371/journal.pcbi.1002172)
Supplement: Text S1 — Model and simulation results description. (PDF) [file pcbi.1002172.s008.pdf]

Model

Figure S1 represents the simulation result of the first model that is described in the main text and characterized in Table 1 and 2. Each time course represents the dynamic change in Lsr-operon concentration over the time period of 500 minutes (~8 hours). AI-2 concentration is varied over the range of 1 to 40 uM (the increase in the concentration is indicated by the direction of the arrow). These simulations follow the experimental result shown in Figure 2B of the main text closely.

We then tested the validity of the model (using the same kinetics rates shown in Table 2) for two of the sub-systems: *lsr*-operon knock-out and *lsrR* knock-out. Similar to the previous case AI-2 concentration is varied over the range of 1-40 uM and dynamic response of the system is plotted as a function of time shown in Figure S2. As it is evidence from these graphs, the system response is significantly lower for the *lsr*-operon knock-out compared to the experimental results shown in Figure 3A (main text). As noted in the main text, the result of Figure 3A was unexpected and we had hypothesized that the proposed model of the system was insufficient to explain the system behavior.

The original model was then modified by the addition of the an *lsr*-operon regulator (REG protein). The second model shown in Figure S3 shows the simulation result of the intact model (left) which corresponds to experimental result of Figure 2 and *lsr*-operon knock-out (right) which corresponds to experimental result of Figure 3A.

Figure S4 summarizes the final modeling results as described in the main text and characterized in Table 3 and 4 (main text). The binding of the *lsr*-regulator to AI-2-P is added to the previous model and this third model now closely follows both the original experimental work of Figure 2B (main text) and the knock-outs presented in Figure 3 (main text).
